# Stress-mediated generation of deleterious ROS in healthy individuals - role of cytochrome c oxidase

**DOI:** 10.1007/s00109-020-01905-y

**Published:** 2020-04-20

**Authors:** Rabia Ramzan, Sebastian Vogt, Bernhard Kadenbach

**Affiliations:** 1 Cardiovascular Research Lab, Biochemical Pharmacological Center, Philipps-University Marburg, Karl-von-Frisch-Strasse 2, D-35043 Marburg, Germany; 2 Department of Heart Surgery, The University Hospital of Giessen and Marburg, Baldinger Strasse 1, D-35043 Marburg, Germany; 3 Department of Chemistry/Biochemistry, Philipps-University Marburg, Hans-Meerwein-Strasse, D-35032 Marburg, Germany

**Keywords:** Stress, Reactive oxygen species (ROS), Mitochondrial membrane potential, Cytochrome c oxidase, Allosteric ATP inhibition

## Abstract

Psychosocial stress is known to cause an increased incidence of coronary heart disease. In addition, multiple other diseases like cancer and diabetes mellitus have been related to stress and are mainly based on excessive formation of reactive oxygen species (ROS) in mitochondria. The molecular interactions between stress and ROS, however, are still unknown. Here we describe the missing molecular link between stress and an increased cellular ROS, based on the regulation of cytochrome c oxidase (COX). In normal healthy cells, the “allosteric ATP inhibition of COX” decreases the oxygen uptake of mitochondria at high ATP/ADP ratios and keeps the mitochondrial membrane potential (ΔΨ_m_) low. Above ΔΨ_m_ values of 140 mV, the production of ROS in mitochondria increases exponentially. Stress signals like hypoxia, stress hormones, and high glutamate or glucose in neurons increase the cytosolic Ca^2+^ concentration which activates a mitochondrial phosphatase that dephosphorylates COX. This dephosphorylated COX exhibits no allosteric ATP inhibition; consequently, an increase of ΔΨ_m_ and ROS formation takes place. The excess production of mitochondrial ROS causes apoptosis or multiple diseases.

## Introduction

Psychosocial stress is known to cause cardiovascular diseases including increased heart rate, high blood pressure, energy mobilization, decreased insulin sensitivity, and endothelial dysfunction [1]. In a multi-cohort study involving 90,164 individuals, the risk of coronary heart disease was 41% higher in individuals with the two work stressors effort–reward imbalance and job strain [2]. Although the molecular basis of these diseases is very complex, the role of oxidative distress [3], in particular increased reactive oxygen species (ROS) production in mitochondria, seems to predominate [4–7]. Furthermore, multiple other diseases like cancer, hypertension, atherosclerosis, ischemia/reperfusion injury, neurodegenerative diseases like Alzheimer’s disease and Parkinson’s disease, rheumatoid arthritis, diabetes mellitus, and mitochondrial diseases have also been related to excessive ROS production in cells [8–11]. But, the detailed molecular sequence of reactions relating psychosocial stress as well as other stressors (hypoxia, xenobiotica, stress hormones, etc.) to increased ROS generation in mitochondria remained unclear. ROS mainly include the superoxide radical anion •O_2_^−^ (half-life ≈ 5 s), hydrogen peroxide H_2_O_2_, and the hydroxyl radical •OH (half-life ≈ 10^−9^ s, formed by the Haber–Weiss reaction: •O_2_^−^ + H_2_O_2_ → O_2_ + •OH + OH^−^, catalyzed by Fe^3+^-ions). In cells, the mitochondrially produced •O_2_^−^ is rapidly converted into H_2_O_2_ by the action of superoxide dismutases present in the matrix and the intermembrane space [12].

Here we describe a molecular mechanism, which represents the missing link between psychosocial stress/other stressors and the generation of cellular distress [3], based on excessive ROS production in mitochondria. This mechanism includes a stress-induced increase of cytosolic calcium, followed by dephosphorylation of cytochrome c oxidase (COX), loss of “allosteric ATP inhibition of COX,” increase of mitochondrial membrane potential ΔΨ_m_, and the formation of ROS.

## ROS have signaling functions

ROS (mainly •O_2_^−^ and hydrogen peroxide H_2_O_2_) are produced in cells by various oxidases that react with molecular oxygen, e.g., NADPH oxidases and xanthine oxidase. Go et al. [13] have listed 31 human cellular oxidases generating H_2_O_2_. Cytochrome c oxidase, however, is the exception and does not release ROS during the reduction of O_2_ to two molecules of H_2_O [14]. ROS are efficiently quenched in normal cells by the antioxidative defense systems including superoxide dismutases, glutathione peroxidase, and catalase [15, 16]. However, small amounts of ROS do have signaling functions in cells, e.g., acting in the maintenance of physiological functions—a process termed redox biology [4]. In a review, the mechanisms and targets of ROS impacting on cell-signaling proteins (NF-κB, MAPKs, Keap1-Nrf2-ARE, and PI3K-Akt), ion channels and transporters (Ca(2+) and mPTP), and modifying protein kinase and ubiquitination/proteasome system have been described [17]. The influence of ROS on metabolic processes such as proteasome function, autophagy, and general inflammatory signaling is discussed by Forrester et al. [18]. In various cancers, ROS have pro-tumorigenic signaling and thus maintain resistance to apoptosis [19]. In normal relaxed cells, low amounts of harmless ROS are maintained by similar activities of its generating and degrading enzymes.

## ROS generation in mitochondria

Almost all energy consumed by aerobic organisms, including heat production and the synthesis of ATP, is produced by fireless burning of food with molecular oxygen (O_2_). ATP is synthesized by oxidative phosphorylation in mitochondria, including the respiratory chain which is composed of complexes I (NADH dehydrogenase), II (succinate dehydrogenase), III (ubiquinol:cytochrome c oxidoreductase or cytochrome bc_1_), IV (COX), and the two-electron carriers: ubiquinone and cytochrome c. Electron transport in complexes I, III, and IV is coupled with the translocation of protons across the inner mitochondrial membrane from the matrix into the intermembrane space creating a membrane potential ΔΨ_m_ and a pH gradient ΔpH_m_ (predominantly ΔΨ_m_), which are used by complex V (ATP synthase) to produce ATP from ADP and phosphate. The oxygen accepting enzyme of the respiratory chain is COX, the rate-limiting step of mitochondrial respiration in vivo [20, 21].

High amounts of ROS are produced in mitochondria under certain conditions, in particular after ischemia and reperfusion in the heart and brain [22]. ROS production occurs in particular at high NADH/NAD^+^ ratios and at a high mitochondrial membrane potential ΔΨ_m_ [23, 24]. In addition to various other sites in mitochondria, ROS are mainly produced from complexes I and III [24–28]. Overproduction of ROS could lead to oxidative damage of lipids, DNA, and proteins [29, 30].

In isolated mitochondria from rat liver or pigeon heart respiring with NAD-linked substrates or succinate, approximately 2% of the total oxygen utilization at state 4 leads to the generation of H_2_O_2_ [31]. At respiratory state 4, all of the phosphate acceptor, i.e., ADP, is converted into ATP and the isolated mitochondria develop a ΔΨ_m_ of 180–200 mV [32, 33]. At the active state 3, where ADP is still available, ΔΨ_m_ has a lower value. Above 140 mV, however, the production of ROS increases exponentially with increasing ΔΨ_m_, as measured in isolated mitochondria [34–36], and with the purified and reconstituted complex III [37]. High ΔΨ_m_ values, accompanied by an increased deleterious ROS production, can be decreased by uncoupler of oxidative phosphorylation. In fact, the use of uncouplers has been proposed as a powerful anti-aging strategy [38] and as a cytoprotective strategy under conditions of oxidative stress including diabetes, drug-resistance in tumor cells, ischemia-reperfusion injury, or aging [39]. These results prove the influence of high ΔΨ_m_ on deleterious mitochondrial ROS production.

In contrast to isolated mitochondria, the mitochondria of relaxed cells in vivo have low ΔΨ_m_ values, i.e., between 100 and 130 mV (references in [40]), at which only very low amounts of ROS are produced [36]. In vivo mitochondria are submerged within 2–10 mM ATP with ATP/ADP ratios of 100–1000 of “free nucleotides,” as calculated from ^31^P-NMR measurements [41]. This means that in vivo the ATP/ADP ratio is always above the half-maximal inhibition of COX activity by the “allosteric ATP inhibition of COX” (see below), which is ATP/ADP = 28 [42, 43]. The frequently cited high number of 2% of total oxygen utilization in mitochondria leading to H_2_O_2_ (ROS) generation [31] corresponds to isolated mitochondria without the allosteric ATP inhibition of COX. The exact value of the low cellular ROS production in vivo, however, is difficult to estimate [23, 44].

## Allosteric ATP inhibition of COX

The unique properties of COX account for its regulatory functions. These are tissue- and developmental-specific isoforms of 6 of the 10 nuclear-encoded “supernumerary” subunits [45, 46], which are tightly bound to three mitochondrially synthesized catalytic subunits I–III [14]; reversible phosphorylation [47–49] and acetylation [50]; binding of various other proteins [51] including the formation of “respirasomes” [52–55]; and reversal binding of small molecules and ions such as ADP or ATP [56], diiodothyronine [57], and calcium or sodium [58].

The kinetic analysis of oxygen uptake of COX at increasing ferrocytochrome c concentrations in the presence of ADP and ATP revealed a sigmoidal shape of the curve with complete inhibition of activity at high ATP/ADP ratios and low amounts of substrate, contrasting the hyperbolic curve in the presence of ADP or without additions [43]. These nucleotides bind to the matrix domain of the transmembraneous subunit IV [42, 43, 59], representing one of the ten ADP-binding sites in COX from the heart, seven of which are exchanged by ATP at high ATP/ADP ratios [60]. The sigmoidal shape of the kinetics indicates cooperativity of two binding sites for ferrocytochrome c (Hill coefficient 2 [43]), suggested to be located at the two monomers of a dimeric enzyme. The X-ray crystal structure of bovine heart COX revealed a homodimeric enzyme [61]. Each COX monomer contains only one cytochrome c binding site [62]. This “allosteric ATP inhibition of COX” keeps ΔΨ_m_ at low values (< 130 mV), due to feedback inhibition of COX activity by ATP at high ATP/ADP ratios. High ATP/ADP ratios already exist at low ΔΨ_m_, because the rate of ATP synthesis by ATP synthase is saturated and maximal at 100–120 mV [63]. Therefore, further increase of ΔΨ_m_ by proton pumping of complexes I, III, and IV of the respiratory chain is inhibited by the ATP inhibition of COX, the rate-limiting enzyme of the respiratory chain in vivo [20, 21]. High ATP/ADP ratios of 100–1000 were measured in vivo by ^31^P-NMR in relaxed cells [41]. The inhibitory effect of ATP on ΔΨ_m_ has also been measured directly in isolated rat liver mitochondria using a tetraphenyl phosphonium electrode [64]. The low ROS production in mitochondria of living cells under relaxed conditions [23] is thus explained by the allosteric ATP inhibition of COX [45, 46, 65, 66].

The allosteric ATP inhibition of COX, however, is not always found with isolated mitochondria and is usually lost during purification of the enzyme [67]. It could be restored by incubation of purified COX with protein kinase A (PKA) and cAMP and can be abolished by incubating again with Ca^2+^ and protein phosphatase 1 [68, 69]. The phosphorylation site at COX was identified towards the intermembrane side of subunit I [68], which contains heme a and the oxygen binding site heme a_3_/Cu_B_ [14]. The reversible switching on of this mechanism by cAMP and switching off by Ca^2+^ was also shown recently using intact isolated rat heart mitochondria [70]. The results with intact mitochondria coincide with the data obtained previously using isolated enzyme [68, 69].

In addition to ATP inhibition of COX activity by the phosphorylated enzyme (postulated phosphorylation site: Ser-441 in subunit I [68]), COX activity is also inhibited by its substrate cytochrome c when it is phosphorylated at serine-47. After dephosphorylation of cytochrome c during ischemia, this attenuation of COX activity is abolished [71].

## Stress and calcium signaling

Calcium represents a universally important messenger in all multicellular life [72]. The cytosolic concentration of calcium in normal resting cells is very low (about 0.1μM) and is more than 10,000 times lower than its concentration in blood plasma (about 2 mM). Numerous extracellular signals from hormones to growth factors are transduced to intracellular [Ca^2+^]i spikes that are amplitude and frequency encoded [73–75]. In addition, they are highly localized within cells [76].

The low cytosolic Ca^2+^ concentration of about 0.1 μM in normal relaxed cells is generally correlated with a low ΔΨ_m_ of 100–120 mV ([references in [41]). Multiple stress signals such as hypoxia, stress hormones, and various chemicals have been shown to increase ΔΨ_m_ to high values via increased cytosolic Ca^2+^ concentrations. This increase of ΔΨ_m_, which occurs often transient, is named “hyperpolarization” of ΔΨ_m_. Numerous studies have demonstrated the hyperpolarization of ΔΨ_m_ by various compounds [77]. The neurotoxic effect of high glucose in diabetes mellitus was studied by Vincent et al. [78] in human SHSY5Y neurons, rat sensory neurons, and Schwann cells. After exposure to 20 mM glucose, an initial transient hyperpolarization of ΔΨ_m_ was measured, followed by an increase of ROS, and finally neuronal death. Mitochondrial hyperpolarization represents an early and reversible step in T cell activation and apoptosis [79]. Transient high ΔΨ_m_ values have also been described by synthetic cannabinoids in human proximal tubule cells [80], by statins (lovastatin and simvastatin), which increased the ΔΨ_m_ in HepG2 and Huh7 human hepatocarcinoma cells and HCC4006 human lung adenocarcinoma cells [81], and by honokiol, which induced in bladder cancer cells and increase of ΔΨ_m_ and ROS formation, and at high doses apoptotic cell death [82]. Hyperpolarization of ΔΨ_m_ was furthermore shown with protamine sulfate [83] and graphene oxide (a marker for air pollution) [84].

All these compounds increase primarily the cytosolic Ca^2+^ concentration, followed by mitochondrial ROS formation and eventually followed by apoptosis and cell death. But also psychosocial stress results in the increase of cytosolic Ca^2+^ concentration, as shown in isolated cardiomyocytes [85], in platelets [86], hippocampal-derived HT22 cells [87], urothelial cells [88], and cardiomyocytes [89]. The neurotoxic effect of glutamate in neurons via the N-methyl-d-aspartat-receptor was related to an increase of cytosolic Ca^2+^ and the formation of ROS [90, 91].

**Fig. 1:**
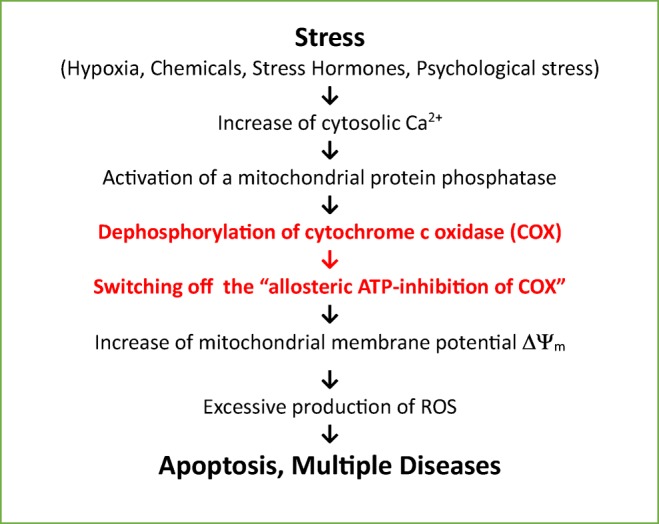
From stress to apoptosis and/or multiple diseases, sequences of molecular reactions

In the sequence of reactions between stress and multiple diseases, shown in Fig. 1, the step between the increase of cytosolic Ca^2+^ and hyperpolarization of ΔΨ_m_ was so far unknown in the current literature [5, 6, 92]. The above-reviewed data explains how stress could induce excessive production of ROS in mitochondria by switching off the allosteric ATP inhibition of COX, which under relaxed conditions, keeps ΔΨ_m_ at low values (below 130 mV). The increase of cytosolic Ca^2+^ concentration by various stress signals from 0.1 to 1–10 μM [73] activates a protein phosphatase which dephosphorylates COX and switches off the allosteric ATP inhibition [68, 69], followed by hyperpolarization of ΔΨ_m_ and increased production of ROS.

From these results, we conclude that the health in all higher organisms is based on the maintenance of a low mitochondrial membrane potential ΔΨ_m_ via the “allosteric ATP inhibition of COX,” which prevents the formation of high and deleterious amounts of ROS. All types of stress signals which increase the cytosolic Ca^2+^ concentration, dephosphorylate COX, and switch off the allosteric ATP inhibition, leading to an increased ROS production via increasing the ΔΨ_m_, thus finally resulting in apoptosis, or in the long run to multiple diseases.

## Near-infrared light suppresses stroke infarct via inhibition of COX activity

The physiological significance of the “allosteric ATP inhibition of COX” was recently verified by experiments of Hüttemann and coworkers, who discovered inhibition of COX activity by near-infrared light of 750- and 950-nm wavelength. The near-infrared light reduced the mitochondrial membrane potential (ΔΨ_m_), attenuated mitochondrial superoxide production (ROS), and in turn neuronal death of cultured HT22 cells following glutamate exposure and oxygen-glucose deprivation [93]. In living animals of a rat stroke model with a longitudinal analysis of brain injury using magnetic resonance imaging, a sustained reduction in infarct volume following ischemic stroke was found after exposure to near-infrared light [94].

These results with living rats coincide with the conclusion derived from results on the allosteric ATP inhibition of COX that attenuation of COX activity prevents the increase of ΔΨ_m_ to values resulting in deleterious mitochondrial ROS production. Another way to decrease ΔΨ_m_ and ROS generation in mitochondria is by using uncoupler of oxidative phosphorylation, which induces a backflow of translocated protons at the inner mitochondrial membrane [95].

## Conclusion

The “allosteric ATP inhibition of COX” is based on the feedback inhibition of COX by ATP, which binds to the matrix side of the “supernumerary” subunit IV. This subunit is lacking in bacteria [96], but it became essential during the evolution of higher organisms, which are characterized by the continuous change between active (state 3 of mitochondrial respiration) and resting state (state 4 of mitochondrial respiration), where ΔΨ_m_ increases. The allosteric ATP inhibition of COX represents an essential mechanism of higher aerobic organisms to avoid the increase of ΔΨ_m_ and thus the formation of deleterious ROS during rest. Without this mechanism, long-living organisms would suffer from various diseases and would die early due to accelerated aging.
